# Impact of a Multimodal Analgesia Protocol in an Intensive Care Unit: A Pre-post Cohort Study

**DOI:** 10.7759/cureus.22786

**Published:** 2022-03-03

**Authors:** Renato Lucas P de Souza, João Abrão, Luís V Garcia, Sofia Vila Moutinho, Ester Wiggers, Andiamira Cagnoni Balestra

**Affiliations:** 1 Anesthesiology, Clinical Hospital of the Faculty of Medicine of Ribeirão Preto of the University of São Paulo (HCFMRP-USP), Ribeirão Preto, BRA; 2 Anesthesiology, University Hospital Center of Algarve, Faro, PRT; 3 Healthcare Scientist, Clinical Hospital of the Faculty of Medicine of Ribeirão Preto of the University of São Paulo (HCFMRP-USP), Ribeirão Preto, BRA; 4 Physiotherapy, Clinical Hospital of the Faculty of Medicine of Ribeirão Preto of the University of São Paulo (HCFMRP-USP), Ribeirão Preto, BRA

**Keywords:** intensive care unit, mortality, mechanical ventilation, opioid analgesics, pain assessment, pain

## Abstract

Introduction

Opioids are the mainstay of pain management in critically ill patients. However, recent attention to their adverse effects in the intensive care unit (ICU) has led to the use of strategies that aim to reduce these side effects. Among these strategies, there are multimodal analgesia protocols, which prioritize pain management and employ a combination of different analgesics to spare excessive doses of opioids and sedatives in continuous infusion.

Objective

The objective of this study is to evaluate the impact of a multimodal analgesia protocol on clinical outcomes and consumption of sedatives and analgesics in two intensive care units.

Methods

We conducted a single-center, quasi-experimental, retrospective, and prospective cohort study comparing clinical outcomes and consumption of sedatives and analgesics before and after the implementation of a multimodal pain management protocol in critically ill adult patients. We included 465 patients in 2017 (pre-intervention group) and 1508 between 2018 and 2020 (post-intervention group).

Results

In the analysis of the primary outcome, there was a significant reduction in mortality between 2017 and 2020 (27.7% - 21.7%, p=0.0134). There was no statistical difference in mechanical ventilation time or concerning the infection rate. Patients who received the multimodal analgesia protocol had a decrease of 24% regarding mean fentanyl intake and a progressive reduction in morphine milligram equivalents (MME) (8.4% - 19%). There was an increasing trend in the use of adjuvant analgesics and morphine in preemptive and therapeutic analgesia.

Conclusion

The implementation of a multimodal pain control protocol significantly reduced morbidity and mortality and the use of opioids in the ICU.

## Introduction

Patients admitted to the intensive care unit (ICU) are vulnerable to severe pain as a result of tissue injury due to serious illnesses, inflammation, major surgeries, and traumas, which require invasive monitoring, mechanical ventilation, immobility, and prolonged hospitalization. Pain, despite being a frequent symptom in the ICU, is underdiagnosed, especially in sedated and intubated patients. It is present in up to 50% of patients at rest and in up to 80% of patients receiving routine care procedures, such as tracheal aspiration, punctures, and drain removal [1]. Up to 77% of patients report moderate to severe pain during ICU stay [2].

Under-treated pain leads to prolonged mechanical ventilation, increased ICU stay, hypoxemia, thromboembolic and pulmonary complications, self-removal of tubes and catheters, violence against caregivers, patient-ventilator asynchrony, immunosuppression, readmission for additional pain control, agitation, myocardial ischemia, delirium, and chronic pain [3].

The main class of drugs used to control pain in the ICU comprises opioid analgesics. Recent guidelines recommend that intravenous opioids be considered as first-line treatment for non-neuropathic pain in critically ill patients [3]. Observational research has shown that opioids are used in more than 80% of ICU patients under mechanical ventilation [4]. However, there are both short-term and long-term risks associated with opioid therapy.

Although the consequences of inadequate pain control are significant, overuse of opioid analgesics and sedatives is often accompanied by unwanted side effects such as hypotension, gastrointestinal hypomotility, paralytic ileus, gastric bleeding, renal dysfunction, immunosuppression, tolerance, hyperalgesia, dependence abstinence syndrome, arousal delay, dependence, risk of developing withdrawal symptoms, prolonged mechanical ventilation and associated problems such as ventilator-associated pneumonia, post-traumatic stress disorder, delirium, unnecessary tests for altered mental status, prolonged ICU stay, decubitus bedsores, nerve compression, respiratory depression, and iatrogenic coma [3].

Multimodal analgesia, due to the significant reduction of opioids, gained great notoriety and evidence in the treatment of postoperative pain [5]. It refers to a pain management strategy that combines non-pharmacological methods (e.g., music therapy, relaxation techniques) and different analgesics with different mechanisms and actions, focused on the central and/or peripheral nervous system, which have an additive or synergistic effect on pain control when compared to unimodal intervention, such as common analgesics, local anesthetics, ketamine, gabapentinoids, and alpha 2 agonists. The combined use of different drugs allows the use of lower total doses of opioids. This, in turn, reduces the number of side effects without impairing patient comfort or preventing rehabilitation [6].

This study examines the effects of implementing a multimodal analgesia protocol in critically ill patients. We imagine that a multimodal approach through a protocol for pain control in critical adult patients may: (1) decrease mortality, time of mechanical ventilation, and infection rate by reducing pain-related complications and overdose of opioid and sedative analgesics; (2) decrease opioid consumption, measured as morphine milligram equivalents (MME), and consumption of sedatives due to increased consumption of adjuvant analgesics.

## Materials and methods

**Figure 1:**
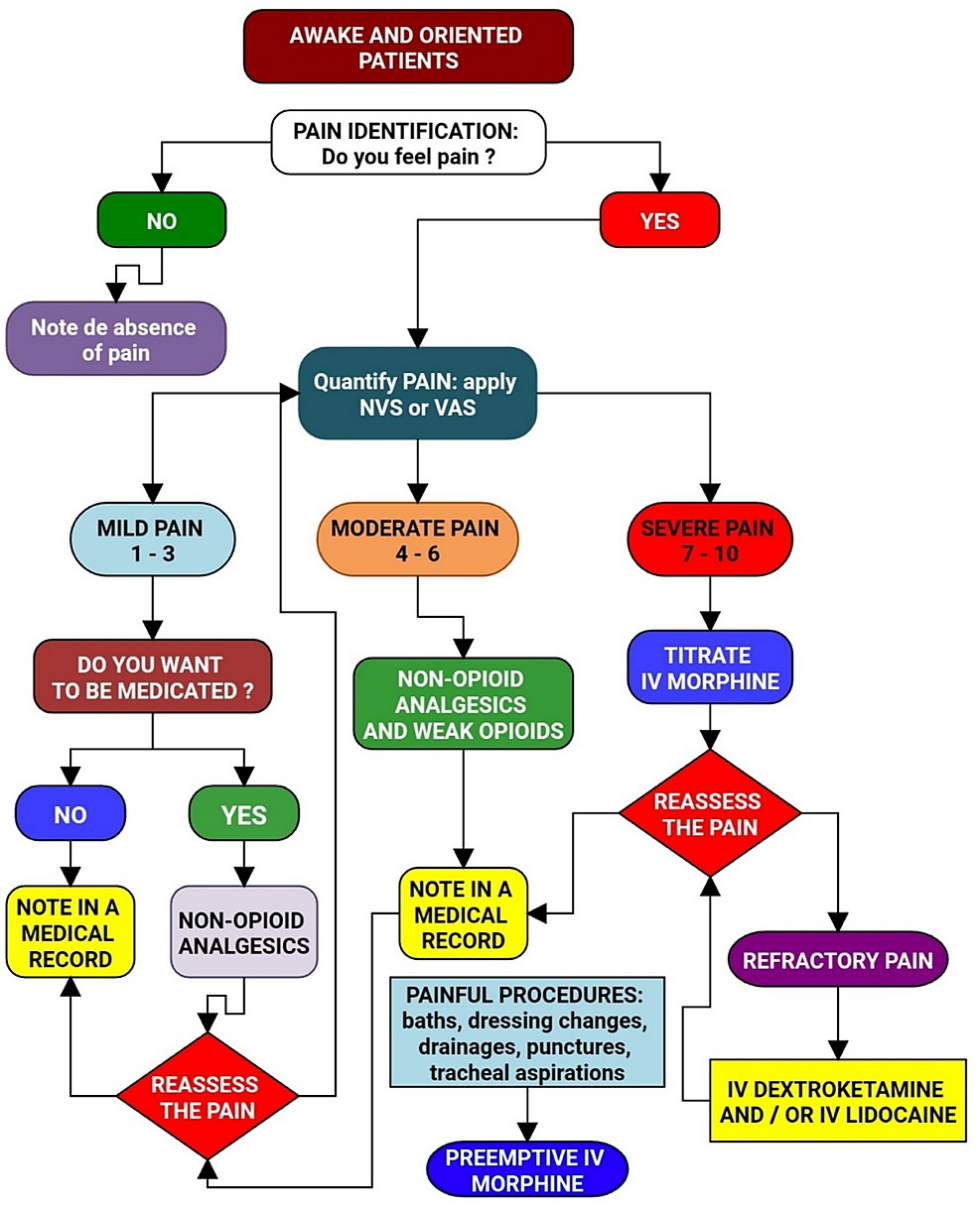
Flowchart of acute pain management in awake and oriented patients Image credits: Renato Lucas P. Souza

**Figure 2:**
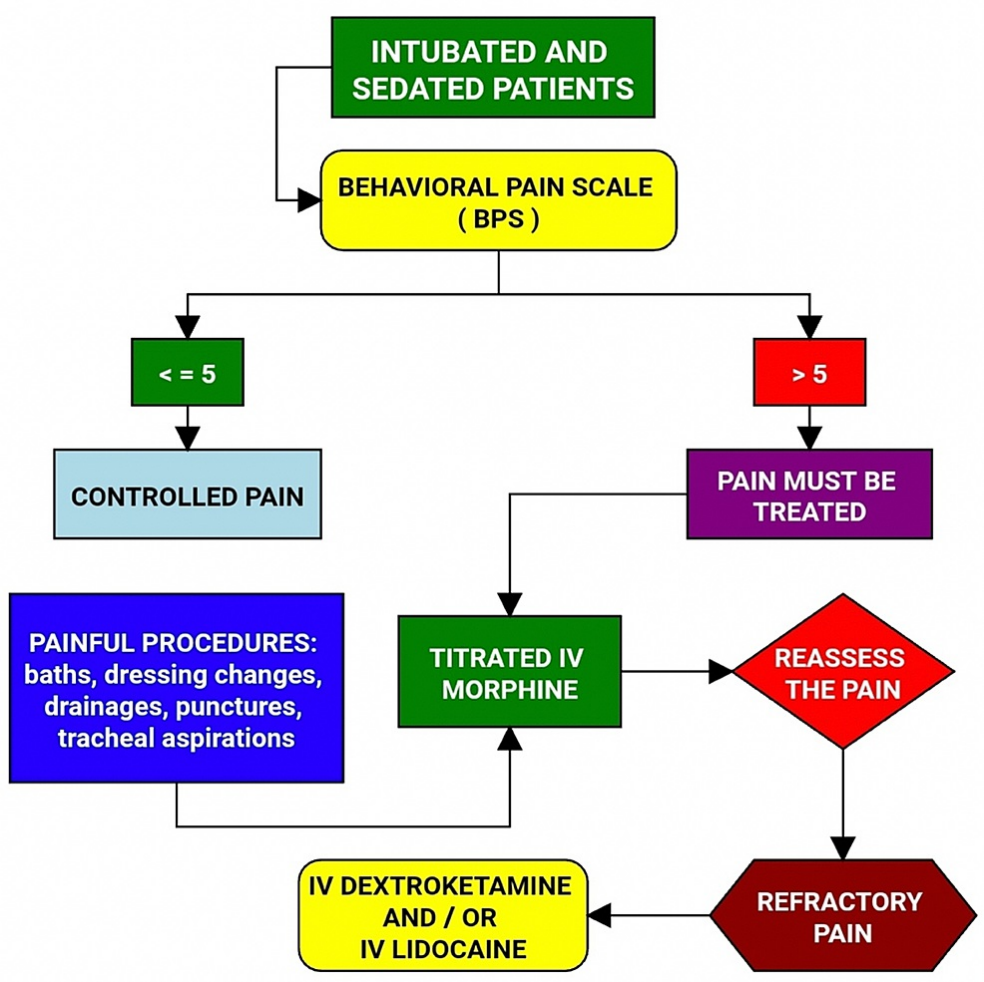
Flowchart of acute pain treatment in intubated and sedated patients Image credits: Renato Lucas P. Souza BPS - behavioral pain scale This should be performed periodically every four hours by a nurse, and should be part of every medical and physical examination

**Figure 3:**
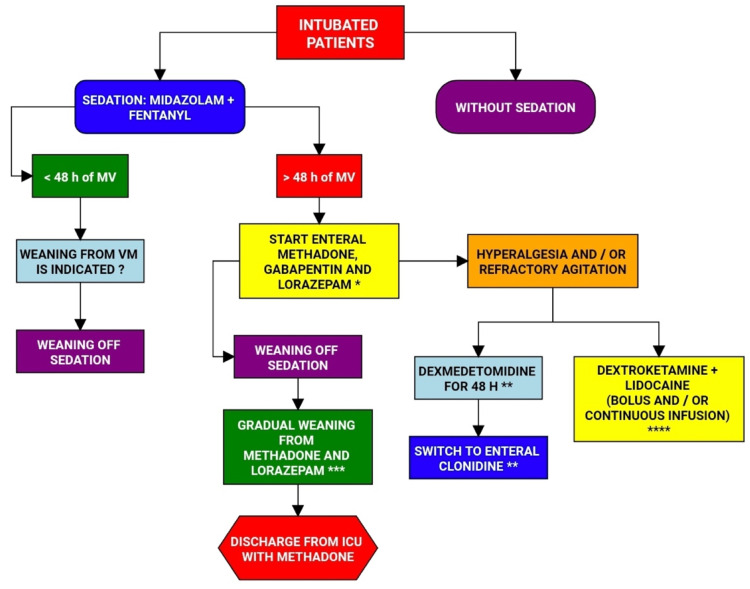
Multimodal analgesia flowchart in intubated patients Image credits: Renato Lucas P. Souza MV - mechanical ventilation * In case of methadone contraindication, start enteral morphine 10mg every four hours ** ICU doctor's decision *** Weaning when indicated, reduce 30% of daily dose every three days **** In case of difficult analgesic control, call the acute pain service

This investigation was designed as a single-center quasi-experimental study conducted in the modality of pre-post cohorts, comparing the impact of an unpublished multimodal analgesia protocol before and after its implementation in two Brazilian ICUs with a total of 14 beds. The protocol below was developed by the researchers of this study and approved by the institution Clinical Hospital of the Faculty of Medicine of Ribeirão Preto of the University of São Paulo (HCFMRP-USP) (Table 1, Figure 1, 2, 3).

**Table 1:** Multimodal analgesia protocol NRS - numerical rating scale, VAS - visual analog scale, BPS - behavioral pain scale,  HCFMRP-USP - Clinical Hospital of the Faculty of Medicine of Ribeirão Preto of the University of São Paulo

Multimodal analgesia protocol	
1	Capacitation and team training: nurses, physiotherapeutic and medical assistants.
2	Systematic and periodic pain assessment of all patients admitted to ICUs by nurses, physicians, and/or physiotherapists, at least every four hours, using validated and standardized pain scales: numerical rating scale (NRS) or visual analog scale (VAS) for patients able to communicate or behavioral pain scale (BPS) for patients who could not be evaluated otherwise.
3	Regular use of analgesics (unless contraindicated by the attending physician)according to pain intensity: Patients with NRS or VAS equal to zero were considered pain-free and did not receive analgesics. Patients with NRS or VAS between one and three were considered with controlled pain and only received common analgesics (dipyrone or paracetamol) if they chose to receive them. In scores between four and six, pain was considered moderate and treated with common venous analgesics (dipyrone) and/or a weak opioid (tramadol). Scores equal to or higher than seven meant severe pain, and the patient was treated with intravenous morphine in doses titrated, according to the patient's response, of 2 to 4mg every 10 minutes to obtain a score lower than or equal to three. In case of pain refractory to morphine, lidocaine bolus 1.5mg/kg and/or dextroketamine 0.2mg/kg and/or peripheral analgesic block were performed (performance by the acute pain team was indicated (Figure 1).
4	Intubated patients and/or those unable to communicate received periodic pain assessments through the BPS scale. Patients with scores lower than or equal to five were considered with adequate analgesic control, and the management was maintained. In persistent evaluations throughout the day with scores above five, multimodal therapy should be readjusted (dose change or introduction of methadone, common analgesics such as dipyrone and paracetamol, and adjuvants such as gabapentin, clonidine, and/or non-steroidal anti-inflammatory drugs). For the treatment of severe acute pain, a strong intravenous rescue opioid should be used, such as morphine (2 to 4mg) or alfentanil (1 to 2 mg) in titrated doses. Reassessment of the score was performed every 10 minutes after the administration of analgesic rescue until reaching a score lower than five. In procedures such as dressing changes, baths, drainages, and punctures, morphine should be administered preemptively and titrated to keep the BPS score lower than five (Figure 2).
5	Sedation and analgesia of adult patients intubated in ICUs of HCFMRP-USP is commonly based on continuous infusion of midazolam and fentanyl. As this drug association was already used as standard sedation in the institution, it was maintained in the protocol to be used initially in newly admitted patients. In newly intubated or tracheostomized patients, with a prognosis of weaning from mechanical ventilation in less than 48 hours, the intensive physician was recommended to wean midazolam and fentanyl (reduction 20 to 50% infusion per day). In the case of intubated or tracheostomized patients, with a forecast of more than 48 hours of mechanical ventilation, it was recommended to introduce methadone 10 mg of 8/8 hours and lorazepam 2 mg of 12/ 12 hours, both by nasoenteral probe (NES). The onset of weaning from methadone and lorazepam, as well as midazolam and fentanyl, was at the physician's discretion, and it was suggested to start with a 30% daily dose decrease every two to three days (Figure 3).
6	In case of hyperalgesia using fentanyl, it was recommended to administer lidocaine in intravenous bolus at a dose of 1.5mg/kg and bolus dextroketamine at a dose of 0.2 mg/kg. Continuous infusion of dextroketamine 0.2 mg/ kg/hour and /or lidocaine 1.5 mg/kg/hour was at the discretion of the intensive physician.
7	In special cases such as agitation, hyperalgesia, or during the weaning process of mechanical ventilation, the use of dexmedetomidine for up to 48 hours was recommended, being replaced by the use of oral clonidine.
8	In cases of contraindication to methadone use as long QT or severe heart disease, morphine 10 mg via SNE every four hours was recommended.
9	In selected cases, such as neurological patients or for midazolam replacement, propofol was used at the discretion of the intensive physician.
10	Patients were discharged from ICUs with a minimum dose of methadone (5 mg every 12 hours) and common analgesics.
11	In case of refractory pain or difficult management, an evaluation of the acute pain service team was recommended.

This research was carried out in the emergency unit of the Clinical Hospital of the Faculty of Medicine of Ribeirão Preto of the University of São Paulo (HCFMRP-USP), having been approved by the Research Ethics Committee of the institution.

All critical adult patients hospitalized between 2017 and 2020 were included in the study, with a uniform distribution of the number of patients each year. Patients with COVID-19 admitted in 2020 were excluded from clinical outcome data analyses, although all received multimodal analgesia according to the protocol. 

Data from all patients was collected and recorded by nursing technicians, nurses, and physiotherapists from the ICU database of the HCFMRP-USP emergency unit, from January 2017 to December 2020. Data from 2017 was collected retrospectively, and data from 2018, 2019, and 2020 were prospectively collected. 

The baseline variables evaluated included: age, gender, and pathological causes of hospitalization. In addition, a simplified acute physiology score, the Simplified Acute Physiology Score 3 (SAPS 3), was calculated in patients during the first 24 hours of hospitalization to analyze the severity of the disease.

The primary outcome was mortality. Secondary outcomes were duration of mechanical ventilation (excluding patients who died), infection rate, and use of sedative and analgesic drugs, both opioid and non-opioid: fentanyl, morphine, morphine, methadone, midazolam, gabapentin, propofol, dexmedetomidine, clonidine, and dextroketamine.

The patients were divided into two groups: the PRE group, composed of patients who were admitted in 2017, before the implementation of the protocol; and the POST group, composed of patients who were admitted between 2018 and 2020, after the beginning of the implementation of the protocol, in January 2018.

Until December 2017, there was no protocoled strategy for pain management and sedation in mechanically ventilated critical patients in the ICUs of the emergency unit of HCFMRP-USP. There were no standardized and validated methods of pain assessment in critically ill patients. Analgesia and sedation, in general, were based on the infusion of fentanyl and midazolam in increasing doses, a common practice in Brazil.

As this study involved all eligible patients (by inclusion/exclusion criteria) during the pre-specified period, a prior calculation of sample size was not performed.

Categorical data was presented as frequency and percentage, while continuous data such as mean were displayed as standard deviation or 95% confidence interval. The correlations between the years for continuous variables were obtained by means of analysis of variance (ANOVA) with post-test (post hoc) of Tukey Honestly Significant Difference (HSD) or, alternatively, Kruskal-Wallis test with Dunn's post-test whenever not possible to meet the assumptions of parametric statistics. All proportions were tested between years, using the Chi-Square test, and the post-test was examined by standardized residual analysis. Additionally, the effect size was reported: generalized Eta^2^ and Eta^2^ based on the statistic H, being considered small (~0.01), medium (~ 0.06) and large (~ 0.14), and Cramer’s V, being considered small (~ 0.2), medium (~0.3) and large (> 0.3). All procedures were performed using Software R version 4.1.1 (The R Foundation, Vienna, Austria).

## Results

**Figure 4:**
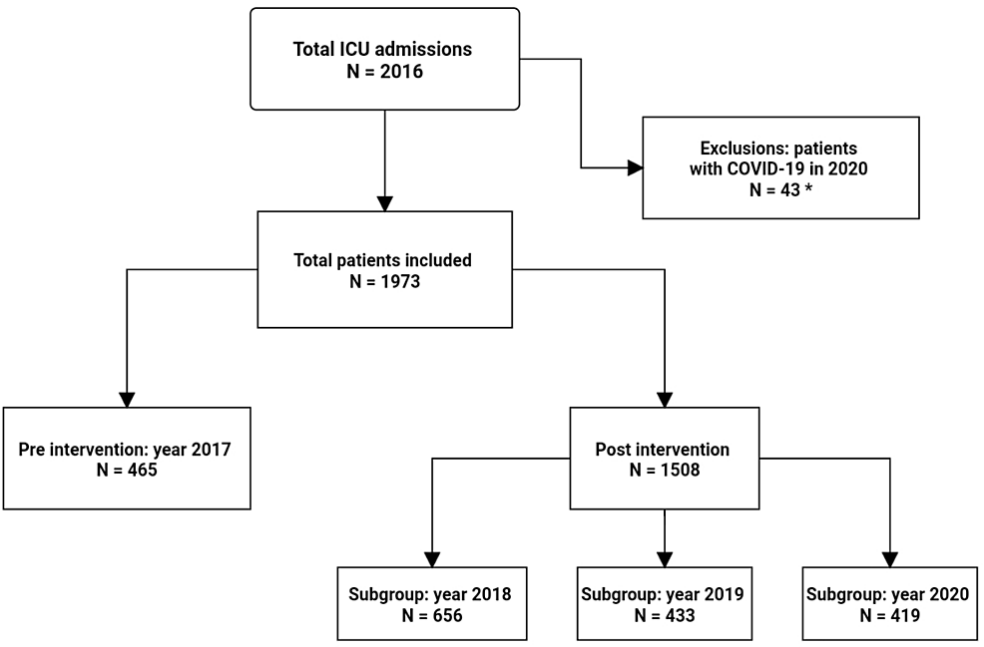
Study design

The sample of this study comprised 1973 patients. There were 465 patients in 2017 (pre-intervention), 656 in 2018, 433 in 2019, and 419 in 2020, totaling 1508 post-intervention patients (Figure 4).

The highest proportion of participants was concentrated in the age group of 50 years, and there was a prevalence of males. The patients of each year presented a similar demographic profile (Table 2).

**Table 2:** Demographic data of the sample, age (years), and gender (n%) SD - standard deviation, n - number of patients in each year, M - male

Demographic data	Year				P-value
	2017 n = 465	2018 n = 656	2019 n = 433	2020 n = 419	
Age, mean ± SD	51.9 (± 18.2)	49.3 (± 17.4)	48.7 (± 17.7)	50.3 (± 17.0)	0.13
Gender: M (n%)	300 (64.5)	430 (65.6)	296 (68.4)	286 (68.3)	0.51

A significant association was found (p<0.0001) between the types of annual occurrences, and the analysis of standardized residuals showed differences in the causes "Other" in 2018 (p=0.0096) and "Cardiovascular" in 2017 (p=0.0187) and 2018 (p<0.0001). There was no difference in age (p=0.13) and gender of participants (p=0.51). (Table 2, 3)

**Table 3:** Frequency observed between occurrence types each year (n%) Number of patients and percentage according to the year and types of occurrence

Year	Neurological	Cardiovascular	Pulmonary	Sepsis	Trauma		Other
2017	170 (36.5)	58 (12.4)	44 (94)	32 (6.8)		82 (17.6)	79 (16.9)
2018	254 (38.7)	31 (4.7)	56 (8.5)	55 (8.4)		101 (15.4)	159(24.2)
2019	168 (38.5)	37 (8.5)	37 (8.5)	39 (9.0)		75 (17.3)	77 (17.9)
2020	152 (36.2)	49 (11.6)	42 (10.0)	28 (6.6)		72 (17.1)	76 (18.1)

**Figure 5:**
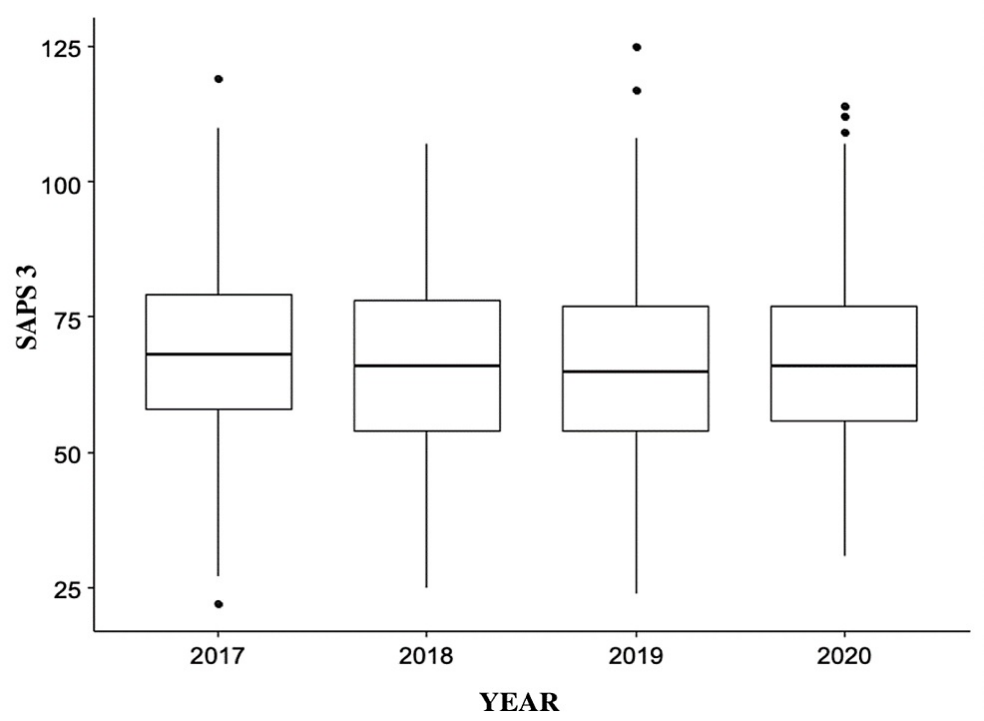
SAPS 3 distribution of patients in the years studied (n=1326) SAPS 3 - Simplified Acute Physiology Score 3

About the score of severity, there was no difference in SAPS 3 value between the years studied (p=0.21) (Figure 5). 

**Figure 6:**
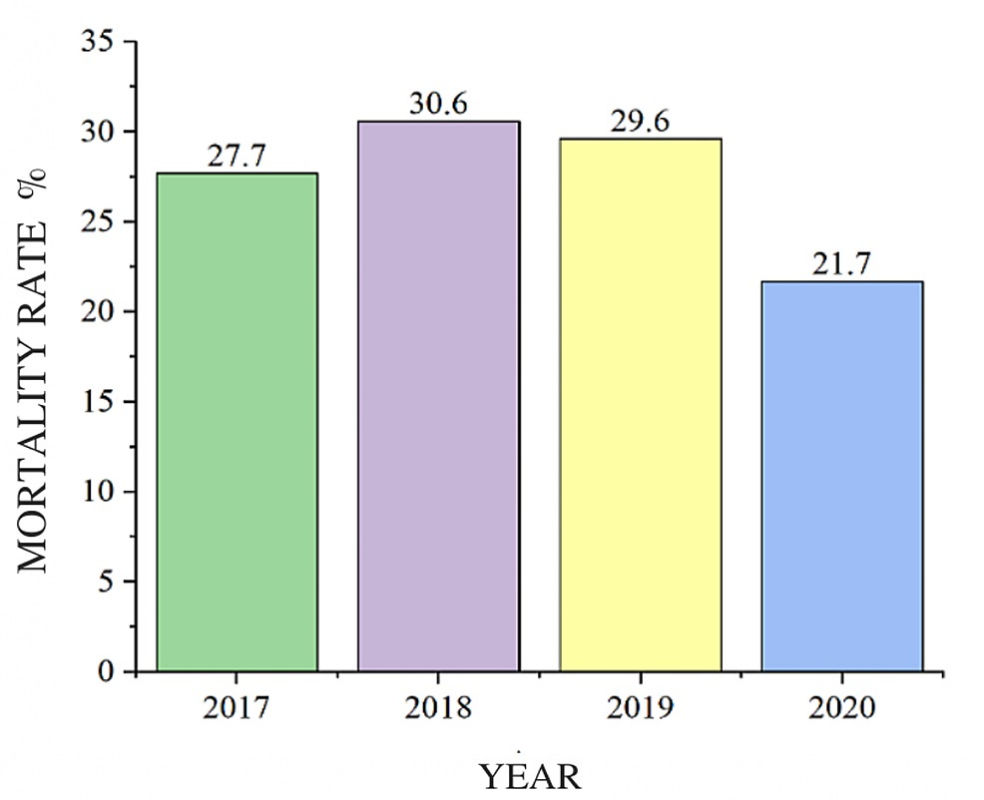
Mortality rate in relation to the years studied

Regarding the primary outcome, the mortality rate fell between 2017 and 2020 (2017 - 27.7%; 2020 - 21.7%). There was a statistical difference in the death rate in relation to the years studied (p=0.0116). The death rate was different in 2020 compared to the others (p=0.0134) (Figure 6).

The total number of patients submitted to mechanical ventilation was 1010 (51.2%). There was no difference between the years in relation to the number of days of mechanical ventilation (p=0.75). 

Regarding the infection rate between the years, there was a difference between 2017 (pre protocol, 24.7%) and the others (after the protocol, mean rate of 21.9%), but no statistical difference in relation to the years studied (p<0.56).

Until 2017, the sedation and analgesia regimen in the ICUs studied was based on the continuous infusion of Fentanyl and Midazolam. After the implementation of the protocol, we observed a progressive decrease in the consumption of fentanyl ampoules in subsequent years (2018, 2019, and 2020) (Table 4).

**Table 4:** Average consumption of medications per patient and total consumption per year n - number, n/pat - number per patient * Included patients with COVID-19

Drug	Presentation	Year							
		2017		2018		2019		2020*	
		n	n/pat	n	n/pat	n	n/pat	n	n/pat
Clonidine	Tablet 150 mcg	995	2.14	776	1.18	1062	2.45	1237	2.95
Dexmedetomidine	Bottle ampoule 200 mcg	605	1.3	1027	1.57	1683	3.89	4933	11.77
Dextroketamine	Ampoule 100 mg					3474	8.02	4750	11.34
Dextroketamine	Ampoule 500 mg	46	0.1	470	0.72	52	0.12	67	0.16
Fentanyl	Bottle ampoule 500 mcg	28915	62.18	24005	36.59	23247	53.69	21355	50.97
Gabapentin	Capsule 300 mg	311	0.67	1148	1.75	2013	4.65	1745	4.16
Lidocaine	2% Ampoule 5 ml	9	0.02	149	0.23	2060	4.76	8187	19.54
Lidocaine	2% bottle ampoule 20 ml	283	0.61	315	0.48	321	0.74	1343	3.21
Methadone	Tablet 5 mg	8276	17.8	17761	27.07	18020	41.62	15332	36.59
Midazolam	Ampoule 50 mg	20319	43.7	18280	27.87	17200	39.72	19323	46.12
Morphine	Ampoule 10 mg 1 ml	596	1.28	1042	1.59	1124	2.6	1567	3.74
Morphine	Tablet 10 mg	824	1.77	192	0.29	113	0.26		
Propofol	Bottle ampoule 200 mg 20 ml	730	1.57	1288	1.96	5071	11.71	4518	10.78

**Figure 7:**
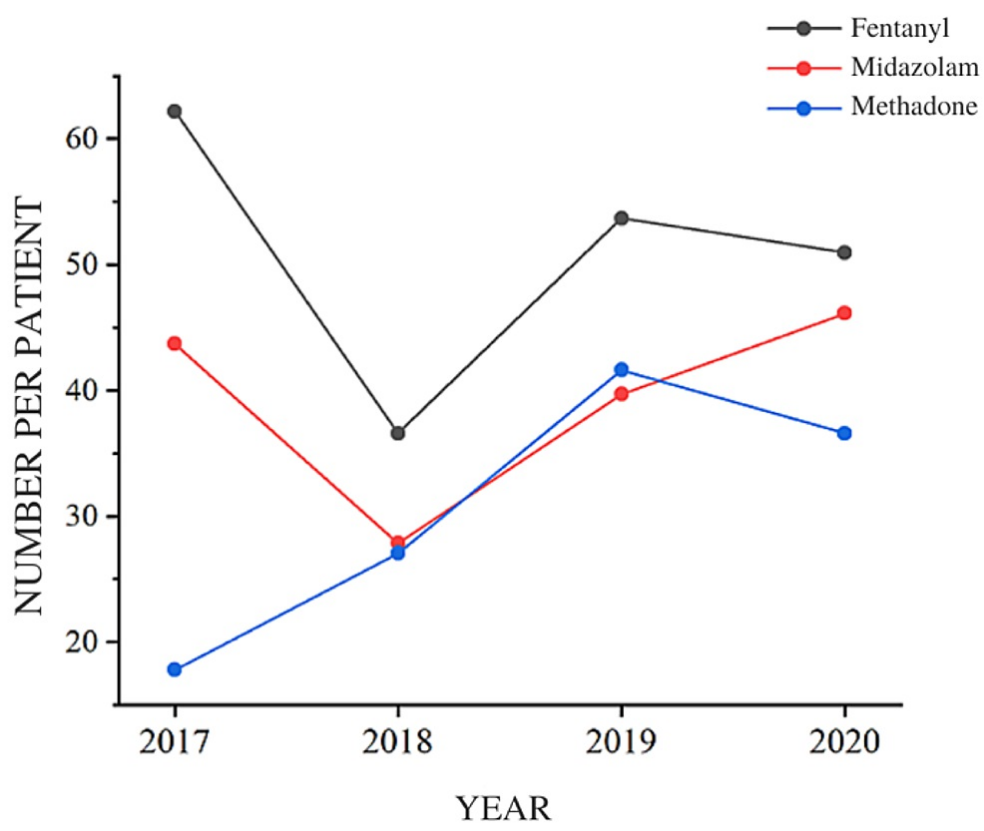
Number of ampoules (Fentanyl, Midazolam) and tablets (Methadone) per patient in each year studied

The difference between the fentanyl intake of the PRE group (28915 ampoules) and the mean fentanyl intake of the POST group (22869 ampoules) was 6046 ampoules (a decrease of 20.9%). The decrease in midazolam consumption was progressive from 2017 to 2019; however, there was an increase in 2020 (when patients with COVID-19 were included in the analysis of drug consumption) (Table 4). Regarding fentanyl intake per patient per year, there was a decrease of 24.27% between the PRE group and the average consumption per patient in the POST group (62.18 - 47.08%) (Table 4; Figure 7). Midazolam had a sharp drop in consumption per patient in 2018 but a progressive increase in subsequent years (Table 4; Figure 7).

Methadone, a crucial medicine of our protocol, had its consumption increased, as predicted, over the years after the intervention. A comparison of consumption per patient per year of the PRE group (17.8 tablets) with the average consumption per patient in the POST group (35.09 tablets per patient) revealed an increase of 97.13% (Table 4; Figure 7).

Regarding the consumption of dextroketamine and lidocaine per patient per year, there was a progressive increase after the intervention. The quantitative consumption of dextroketamine per patient was increased by 2428% in 2020 as compared to 2017 (pre-intervention). Lidocaine at 2% consumption per patient increased by 1316,26% in 2020, compared to 2017 (pre-intervention) (Table 4)

The consumption of propofol, clonidine, gabapentin, and dexmedetomidine was also analyzed (Table 4). All these medications had a very low consumption in the pre-intervention year. Regarding propofol, comparing the consumption of ampoules per patient in the PRE group with the mean consumption per patient in the POST group, there was an increase of 509.10%. In the third year after the intervention (2020), there was an increase of 686.62% in the consumption of propofol ampoules per patient in relation to the PRE group. Regarding clonidine, comparing the consumption of tablets per patient in the POST group with the mean consumption per patient in the POST group, there was an increase of 215.57%. In the third year after the intervention (2020), there was an increase of 137.85% in clonidine intake per patient compared to the PRE group. The average consumption of gabapentin capsules per patient in the POST group increased by 525.37%, compared to the PRE group. In the third year after the intervention (2020), there was a 620.89% increase in gabapentin consumption per patient, compared to the PRE group. The average consumption of dexmedetomidine ampoules per patient in the POST group increased by 430.76%, compared to the PRE group. However, the increase in consumption was very significant in the third year after the intervention (2020), with an increase of 905.38% in the consumption of dexmedetomidine per patient in relation to the PRE group (Table 4).

There is a wide variety of opioids with different potencies. We used morphine milligram equivalents (MME) as the consumption metric for opioid analgesics. MMEs are responsible for the potency of the opioids consumed. There was a progressive decrease in total consumption of MMEs from 2017 to 2020 (Table 5).

**Table 5:** Total consumption per year of morphine milligram equivalents MMEs - morphine miligram equivalents * Included patients with COVID-19

MMEs	2017	2018	2019	2020*
mg	3112106	2851063	2755990	2519814

**Figure 8:**
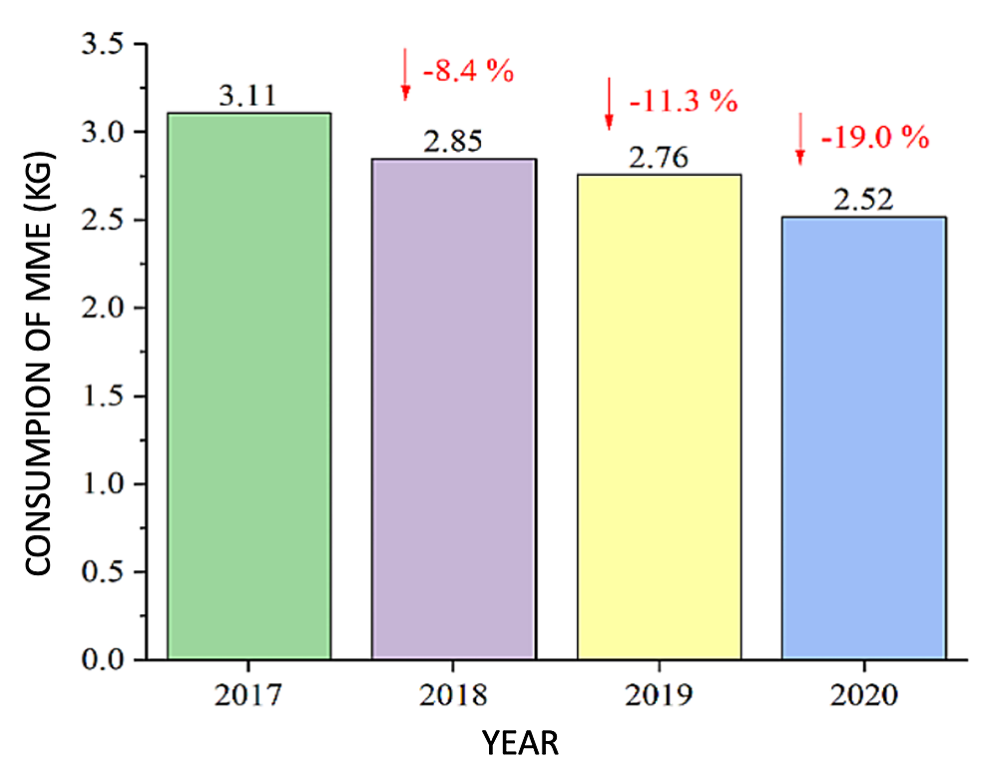
Total consumption of MMEs each year MMEs - morphine miligram equivalents

**Figure 9:**
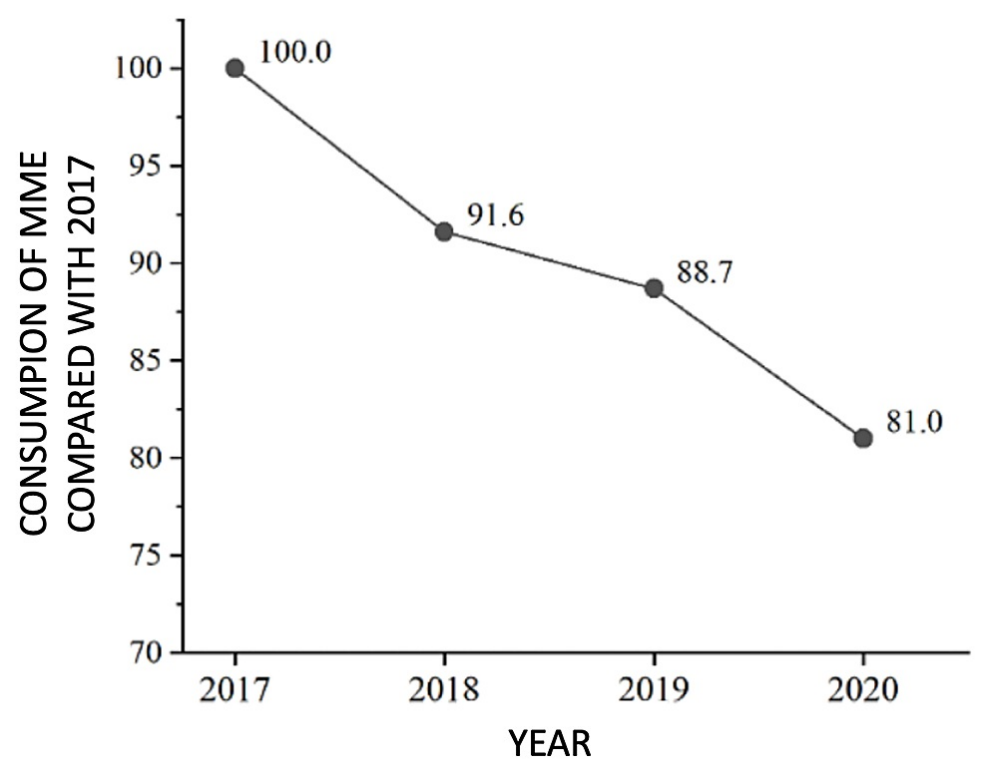
Fall in MMEs consumption since 2017 (pre-intervention) MMEs - morphine miligram equivalents

In relation to the pre-intervention year (PRE group), there was an 8.4% drop in the consumption of MMEs in the first year after the intervention (2018), followed by a drop of 11.83% in 2019 and 19% in 2020 (Table 5, Figure 8, 9).

In our protocol, morphine was used for acute pain treatment and pre-procedure preemptive analgesia. Morphine consumption per patient decreased in the first year after intervention (2018) but increased considerably in subsequent years (2019 and 2020). The increase in morphine consumption was very significant in the third year after the intervention (2020), with an increase of 122.62% in consumption per patient in relation to the PRE group.

## Discussion

The present study, through a multimodal, preemptive, and preventive analgesic approach, using a validated behavioral scale for pain assessment (BPS), showed that an intervention with the objective of improving pain management can generate favorable results. This way, we value the possible benefits of the management of protocoled multimodal analgesia. Our main finding was the decrease in the mortality rate in the 2020 subgroup compared to the other years, including the decrease by 6% compared to the pre-protocol subgroup of 2017 (2017: 27.7%; 2020: 21.7%) (p=0.0134). In a prospective pre-post study methodology, Skrobik et al. [7] found similar results in relation to mortality. In addition to the significant decrease in the mortality rate, they also found a decrease in delirium, time of mechanical ventilation, and length of ICU stay in the post-analgesia and sedation protocol group. Although we did not study the occurrence of delirium in our ICUs, the decrease in its incidence may be a possible causal factor for the decrease in mortality found in our study [8, 9]. Pain and sedative-induced coma, such as benzodiazepines, are powerful risk factors for the occurrence of delirium in the ICU, and delirium, independently, decreases survival [8]. Unlike the protocol of our study, the protocol of Skrobik et al. included only opioids, propofol, benzodiazepines, paracetamol, and anti-inflammatory drugs [7].

Critically ill adults experience moderate to severe pain at rest and during standard care procedures [1,10]. Until the implementation of our protocol, European Union ICUs did not perform routine pain assessment and preemptive analgesia. The prevalence, intensity, and risk factors of pain related to these procedures were not well-known until the prospective, cross-sectional and multicenter study in 192 ICUs from 28 countries [8,9]. All 2457 patients who were able to speak or communicate in some way (65.1% of the total) reported mild pre-procedure pain intensity but experienced a significant increase in pain intensity during the procedure for all procedures. These findings emphasize the importance of pre-procedure pain assessment and preemptive analgesia, when appropriate, for procedures known to cause pain. In our study, we found an increase in the consumption of morphine and other analgesic adjuvants (dextroketamine and lidocaine) due to our protocol contemplating preemptive analgesia and the treatment of acute pain.

Although pain assessment is strongly recommended by the guidelines, its broad adoption is not universal [2]. Even though we did not analyze the degree of protocol adhering and pain assessment rate through the BPS scale, we noticed an increase in the consumption of morphine and adjuvant analgesics after the intervention. In a recent cross-sectional study of 45 ICUs in the UK, physicians did not document pain assessment in nearly two-thirds of patients; nurses stopped documenting pain assessment in 28.6% of patients [11]. Luetz et al. found better results in a European multinational survey: 81 out of 101 ICUs reported pain assessment, but only 24 used a validated scale for patients unable to communicate [12]. A Dutch national study confirmed these findings: broad adoption of pain assessment scales for patients able to communicate, but low use of behavioral pain scales [13].

Patient-focused analgesia algorithms are multidisciplinary, including the training and development of physicians, nurses, physiotherapists, and pharmacists. Implementing algorithms in ICUs is a challenging process for which sufficient resources should be allocated. In a prospective, observational, non-randomized study, the implementation of a multidisciplinary protocol of analgesia, sedation, and delirium lasted 13 months [7]. In the present study, the introduction of the protocol was performed only after the structured education of all team members who cared directly for the patient between October 2017 and January 2018. The training of physicians, patients, nursing technicians, physiotherapists, and pharmacists was provided by lectures given by the researcher on predetermined service days. In addition to these members, a nursing professional certified in pain study was fundamental in the education and implementation process. Marked changes that a protocol causes in individual practice require time to be absorbed, adapted, and implemented by the professionals involved. This adaptation time may impair the adaptation and justify the differences in the results after implementation of the protocol in the subgroups of patients from the years 2018, 2019, and 2020. This study did not analyze the rate of professional participation in the protocol. However, protocolization seemed to reduce variability in practice, as reflected by changes in drug consumption in the period studied.

Ideal analgosedation in patients who are victims of severe trauma on mechanical ventilation is often challenging. ICUs from the HCFMRP-USP Emergency Unit constantly receive trauma patients. During the period of this study, severe trauma was the main reason for ICU admission in 330 patients (16.7%). Our study did not specifically analyze this subgroup, but Robinson et al., in a retrospective pre-post study with 143 critically ill trauma victims, found shorter mechanical ventilation and hospitalization times in the group that received multidisciplinary analgesia, sedation, and delirium protocol [14]. In a recent pre-post cohort study, implementation of a multimodal pain management strategy significantly reduced opioid use in critically ill trauma patients [15].

In our study, 51.2% of the patients underwent mechanical ventilation. Although we observed a decrease in opioid consumption over the years and increased consumption of adjuvant drugs, in relation to the time of mechanical ventilation, there was no significant difference between the years studied and between the PRE and POST subgroups of the protocol. Regarding the infection rate, we demonstrated a decrease of 2.9% between the PRE group and the mean of the three subgroups POST, but without statistical significance. Published sedation protocols have been tested in controlled clinical trials, often demonstrating benefits such as shorter duration of mechanical ventilation, reduced length of ICU stay, and/or better management of sedation, compared to the usual treatment [16]. Chanques et al., in a pre-post study, showed that the systematic evaluation of pain levels, sedation, and agitation by nurses through scales, with rapid response from a physician in case of need, decreased the incidence and intensity of pain and agitation in ICU patients [17]. This improvement in pain and agitation control was associated with a shorter duration of sedation, mechanical ventilation, and fewer nosocomial infections. These results can be explained by a better correspondence between analgesics, sedatives, and the needs of patients for these drugs. However, the decrease in the duration of mechanical ventilation and the rate of infections should be explained especially by the physician's education, which encourages them to discontinue or decrease the administration of analgesics or sedatives in the absence of pain or agitation.

Our protocol implemented changes in the routine of the ICUs studied, requiring systematic and periodic evaluation of pain through the BPS scale. Our study showed that a multimodal pharmacological intervention aimed at improving pain management may reduce opioid consumption, according to suggestions from the Society of Critical Care Medicine guidelines of 2018 that support the use of multimodal pharmacotherapy as a component of an analgesia approach to save and/or minimize opioids and sedatives. According to the guidelines, the multimodal analgesia strategy probably improves pain control, reduces opioid consumption, and improves patient-centered outcomes [2]. Previous studies have demonstrated the efficacy of systematic pain assessment in critically ill patients. In a large cohort study, pain assessment was associated with a shorter duration of mechanical ventilation and a shorter length of stay in the ICU [18]. Pre-post studies confirmed these findings [7,19]. In addition, the improvement in the systematic evaluation of pain with validated scales promotes an increase in the use of non-opioid multimodal pharmacotherapy [17,18]. Our study, with a similar design, did not show a reduction in the duration of mechanical ventilation, even though there was a decrease in the consumption of fentanyl, an opioid with a long half-life when used in continuous infusions (half-life highly dependent context).

The use of adjuvant drugs, in addition to opioids, can help provide effective analgesia while minimizing unwanted side effects. There is evidence for the use of non-opioid analgesics in other acute care environments, such as emergency departments and post-anesthetic treatment units, but the efficacy and degree of use of non-opioid analgesics in the ICU are not well-documented [20,21]. Our protocol included the use of several non-opioid analgesics. A systematic review and meta-analysis with 34 eligible studies showed that the use of any adjuvant, in addition to an opioid, led to reductions in pain scores reported by the patient and in opioid consumption over 24 hours [22]. Among the individual drugs, reductions in opioid use were demonstrated with dexmedetomidine, nefopam, non-steroidal anti-inflammatory drugs (NSAIDs), paracetamol, carbamazepine, ketamine, and tramadol. Reductions in the duration of mechanical ventilation during ICU stay were shown with the use of any adjuvant analgesic, although this was based on very low evidence of certainty, and the magnitude of the difference was of minimal clinical importance. No individual adjuvant medication showed an effect on the duration of mechanical ventilation or on ICU stay. The results of Wheeler et al. are consistent with another recent meta-analysis that also summarize the use of non-opioid analgesics for ICU patients [22,23].

There are few studies to date that suggest positive impacts on critical patient care, such as the reduction of opioid consumption with the use of multimodal analgesia protocols in the ICU. Nevertheless, protocols that, at least, required systematic evaluations with validated scales of pain and sedation consistently reduced opioid and sedative consumption [18]. Our study showed that a multimodal pharmacological intervention aimed at improving pain management in ICU can reduce opioid consumption (MME) by 8.4 to 19.1% from a previous baseline level and lead to a sustained trend of lower use both in the short and long term (at least up to 3 years after the intervention). This occurred in parallel with the increased use of adjuvant analgesics (dexmedetomidine, clonidine, gabapentin, dextroketamine, and lidocaine), without any deleterious effect observed in the analyses of clinical outcomes in the pre-post cohorts. The intervention also significantly reduced the use of fentanyl with a mean post-protocol savings of 2520 ampoules per year (2018, 2019, and 2020) and a mean reduction of 15.1 ampoules per patient. Our study did not compare the cost of medications each year due to inflation in the period studied, different quotations between years, or the increase in drug prices during the COVID-19 pandemic. In a Brazilian single-center retrospective pre-post study, a pain management protocol characterized by routine pain assessment, increased use of dipyrone and diluted fentanyl solution, substantially reduced the use of fentanyl in the ICU, and demonstrated shorter duration of mechanical ventilation [24]. In our protocol, the use of common analgesics, such as dipyrone and paracetamol was stimulated, but it was not analyzed in the study. 

The pre-post study of Skrobik et al. demonstrated superior analgesia and significantly lower mean doses of opioids (four times lower) in the post-protocol of analgesia sedation and delirium group [7]. Both cohorts received an equivalent amount of paracetamol and anti-inflammatory drugs. Average doses of MME decreased from 45.17 to 9.90. Like our study, a pre-post cohort evaluated the effects of multimodal analgesia on opioid needs in critically ill patients with severe trauma. The mean cumulative dose of MME was significantly lower in the post-intervention period. Patients who received three or more multimodal agents (gabapentin, anti-inflammatory drugs, central muscle relaxers, and/or peripheral blocks) had lower cumulative MME compared to those who received one to two or 0 multimodal agents, without comfort impairment [15].

The practice of analgosedation in the ICU, that is, first using analgesia instead of sedation, are new paradigms in the management of patients on mechanical ventilation and form the basis of the current practice of sedation. The results of a retrospective pre-post study demonstrated that an analgosedation protocol was associated with a lighter level of sedation than a previous sedative-based strategy. In addition, the authors found a shorter duration of mechanical ventilation time and length of stay in the ICU. The study also demonstrated a significant decrease in the use of sedative agents in continuous infusion (fewer benzodiazepines and more dexmedetomidine), a significant increase in opioid analgesics, and a potential reduction in the costs of associated drugs [19]. In our study, however, we found a decrease in the consumption of post-intervention fentanyl. On the other hand, there was an increase in the consumption of other opioids such as methadone, morphine, and other adjuvants, proving a certain degree of protocol adherence. After the implementation of the protocol, professionals were more concerned about reducing the side effects of excessive use of fentanyl and midazolam (common practice before the intervention). The increased consumption of dextroketamine, lidocaine, and morphine after intervention also suggests greater use of preemptive analgesia and treatment of moderate to severe acute pain after the intervention. The results of a French cohort study demonstrated that the use of multimodal analgesia (with analgesics paracetamol or nefopam) in critically ill patients under mechanical ventilation may decrease sedation and delirium and, at the same time, decrease opioid use and opioid-related side effects. Patients who received multimodal analgesia were also more likely to have fewer organic failures and received fewer hypnotics, compared to patients who received only opioids [25]. In another retrospective cohort study, which meets our results, burned patients treated with multimodal analgesia in the ICU were compared to those burned treated only with opioids. The use of multimodal analgesia significantly reduced the equivalent dose of cumulative opioids and did not compromise pain control [26].

Pain management is not only a strategy to improve patient comfort and outcomes, but also a means of reducing sedation. The decrease in the use of sedatives can also be a useful intervention in reducing opioid consumption, provided that the appropriate assessment of pain precedes it [27]. Our findings showed a decrease in the consumption of midazolam after intervention with a subsequent increase in 2020, plausibly explained by the inevitable inclusion of 43 patients with COVID-19 in this analysis. Patients with severe COVID-19 have abnormally high needs for sedative medications, as well as the use of combined sedation therapies and strategies, leading to a major challenge [28]. On the other hand, there was an increase in the consumption of propofol and dexmedetomidine after the intervention. These findings are in line with the latest guidelines that recommend that sedatives such as propofol or dexmedetomidine are preferable to benzodiazepine sedatives (midazolam or lorazepam) in mechanically ventilated critical adults as well as the use of mild sedation (versus deep sedation) due to improved short-term outcomes, such as ICU admission, duration of mechanical ventilation, and delirium [2].

The reduction in opioid consumption in MME was predicted in our study. While Faust et al. showed that a better pain assessment increased the use per patient of fentanyl, others revealed opposite results [7,19]. Our findings corroborate the latter. Although the association between a better assessment of pain and decreased opioid use is counterintuitive, there are some possible reasons to explain this outcome. First, routine pain management strategies focus on pain assessment. Therefore, as the opportunities for evaluation increase, there is also a reassessment of doses. In the period before the implementation of our strategy, physicians and nurses started the infusion of high doses of fentanyl and midazolam, as recommended in previous guidelines and without periodic standardized reassessments. This approach could mean the use of fentanyl as a sedative, which only potentiates its prolonged effects [29]. Our results show a progressive decrease in fentanyl consumption over the post-intervention years, a decrease in morphine consumption per patient in the first year after the intervention, and an increase in subsequent years, suggesting lower use of fentanyl as a sedative, greater adoption of preemptive analgesia practices and management of acute pain in the previous two years. Second, a multimodal approach to analgesia could save opioid consumption. The use of adjuvants increased after the implementation of our protocol, with a multimodal approach to pain management. Many studies on critically ill patients have shown that the use of non-opioid analgesics decreases opioid consumption, with no differences in terms of pain scores [7,15,19,26]. The use of non-opioid analgesics also allows for lower levels of sedation and reduces the time until extubation [25]. A third reason that we believe may have played a role in reducing opioid use was the possible reduction of opioid tolerance, which was one of the objectives of our protocol. Long-term opioid use leads to tolerance, i.e., less susceptibility to opioid effects, which may result in the need for larger and more frequent doses to achieve the same analgesic effect. Tolerance to opioids can be observed during all types of critical diseases; the magnitude, however, seems exaggerated in patients who have suffered major trauma (e.g., burns), in patients requiring prolonged mechanical ventilation, and in pediatric patients. The development of tolerance is due in part to the large doses necessary to control pain in these critically ill patients. The use of methadone early, as well as the use of adjuvants (dexmedetomidine, clonidine, ketamine, lidocaine, gabapentin), and a decreased use of midazolam attenuate fentanyl tolerance [30].

Limitations of the Study

Among the limitations of this study, we can highlight the following: (1) it was an observational, non-randomized study. The pre-post design was chosen to better simulate real-life clinical situations due to concern that a randomized model would be contaminated by culture change over time and because observational studies can provide valuable evidence, even when compared to randomized trials. These findings may show only association and no causality. The design of this study prevents any attribution of causality between analgesia management and mortality, even comparing homogeneous populations. At the very least, our findings suggest that the protocol does not pose any risk. The association of the protocol with a decrease in mortality should be validated with a randomized clinical trial. (2) The variables chosen were limited to only those available before the intervention. It was merely possible to include patients of the year 2017 in the PRE group due to the lack of data concerning the previous years. (3) We adjusted the analyses to compare the two periods, but we could not do it for all possible confounding factors; other variations in care and secular tendency may also have contributed to the observed results, especially mortality, infection rate, and duration of mechanical ventilation (MV). Nonetheless, the substantial reduction in opioid consumption is a clinically significant result that may have led to a significant reduction in complications and unnecessary expenses and may have had an impact on outcomes. (4) We do not have pain measurements available for the purposes of this study, so we cannot prove that patients had adequate pain control. However, the entire ICU team was trained to adequately assess pain, with special attention to the BPS scale and pre-procedure analgesia. (5) Our results are from a single-center study and may not be generalizable, although these findings may help other centers to evaluate their pain management protocols, which may have an impact on clinical outcomes. (6) We were not able to evaluate the side effects of opioids with our methodology. (7) The presence of multiple pharmacological interventions limits the assignment of results to one intervention or another. (8) We did not evaluate the incidence of delirium, agitation, and level of sedation; however, a multimodal analgesia protocol probably reduces iatrogenic coma, delirium, and agitation of mechanically ventilated patients, and further studies are needed to confirm these hypotheses. 

## Conclusions

A multimodal analgesia protocol in the ICU, characterized by routine evaluation of pain with validated behavioral scale, increased use of adjuvant analgesics, and use of preemptive analgesia substantially, decreased the use of fentanyl and the consumption of MMEs in the intensive care unit. This strategy was associated with increased use of adjuvant analgesics (ketamine, gabapentin, dexmedetomidine, and clonidine) and propofol and a significant decrease in mortality three years after the beginning of the intervention. There were no changes regarding mechanical ventilation time and infection rates. The protocol developed in our study proved to be efficient for the study proposal, and our results are consistent with previous research. Randomized clinical trials are needed to confirm these findings.
